# Retinal involvement in an acute lymphoblastic leukemia

**DOI:** 10.11604/pamj.2019.32.192.9648

**Published:** 2019-04-18

**Authors:** Fouad Chraibi, Idriss Andaloussi Benatiya

**Affiliations:** 1 University Allal Ben Abdellah, University Hospital Hassan II, Fez, Morocco

**Keywords:** Retinal infiltration, lymphoblastic leukemia, papilledema

## Image in medicine

We report a case of a patient who presents for ophthalmic complaints revealing systemic acute lymphoblastic leukemia. The eye fundus examination shows bilateral papilledema, retinal hemorrhages and diffuse white retinal infiltration. After chemotherapy, there was noticeable improvement of visual acuity and important regression of papilledema. Ocular involvement in leukemia is considered as a central nervous system lesion. It requires, therefore, a proper treatment including corticosteroids, chemotherapy and radiation treatment of the central nervous system.

**Figure 1:**
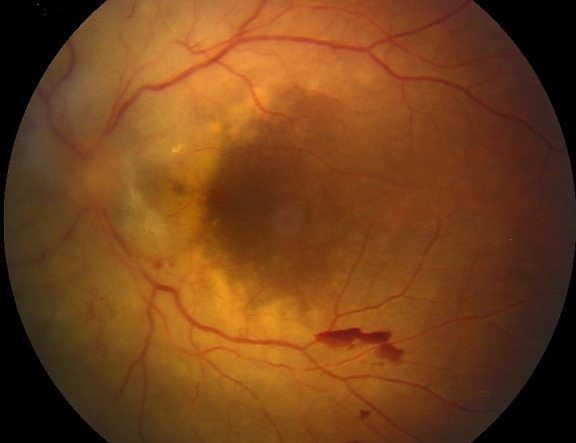
Eye fundus aspect

